# A new experimental rat model of nocebo‐related nausea involving double mechanisms of observational learning and conditioning

**DOI:** 10.1111/cns.14389

**Published:** 2023-08-07

**Authors:** Yu Zhang, Wanbin Huang, Zhengming Shan, Yanjie Zhou, Tao Qiu, Luyu Hu, Liu Yang, Yue Wang, Zheman Xiao

**Affiliations:** ^1^ Department of Neurology Renmin Hospital of Wuhan University Wuhan Hubei Province China; ^2^ Central Laboratory Renmin Hospital of Wuhan University Wuhan Hubei Province China

**Keywords:** animal model, cholecystokinin, nausea, nocebo, placebo, vomiting

## Abstract

**Aim:**

The nocebo effect, such as nausea and vomiting, is one of the major reasons patients discontinue therapy. The underlying mechanisms remain unknown due to a lack of reliable experimental models. The goal of this study was to develop a new animal model of nocebo‐related nausea by combining observational learning and Pavlovian conditioning paradigms.

**Methods:**

Male Sprague–Dawley rats with nitroglycerin‐induced migraine were given 0.9% saline (a placebo) or LiCl (a nausea inducer) following headache relief, according to different paradigms.

**Results:**

Both strategies provoked nocebo nausea responses, with the conditioning paradigm having a greater induction impact. The superposition of two mechanisms led to a further increase in nausea responses. A preliminary investigation of the underlying mechanism revealed clearly raised peripheral and central cholecystokinin (CCK) levels, as well as specific changes in the 5‐hydroxytryptamine and cannabinoid systems. Brain networks related to emotion, cognition, and visceral sense expressed higher c‐Fos‐positive neurons, including the anterior cingulate cortex (ACC), insula, basolateral amygdala (BLA), thalamic paraventricular nucleus (PVT), hypothalamic paraventricular nucleus (PVN), nucleus tractus solitarius (NTS), periaqueductal gray (PAG), and dorsal raphe nucleus‐dorsal part (DRD). We also found that nausea expectances in the model could last for at least 12 days.

**Conclusion:**

The present study provides a useful experimental model of nocebo nausea that might be used to develop potential molecular pathways and therapeutic strategies for nocebo.

## Graphical abstract

Different processes, such as Pavlovian conditioning and observational learning, can both cause nocebo nausea in Rats. Emotional, cognitive, and visceral sense brain areas were possibly activated, indicating the involvement of complex neural loops.

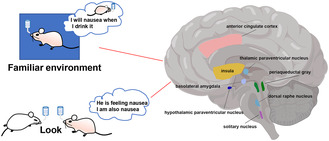

## 1 INTRODUCTION

The nocebo, as the inverse of the placebo, refers to an inactive ingredient that can produce harmful responses.^1^ These bothersome nocebo events derive from the use of the intervention itself but not from real pharmacological action, and have been proven to link with negative expectations, previous learning experiences, and conditioning.^2^ Up to 25%–80% of patients reported adverse events after receiving a placebo in randomized controlled trials, and 2%–10% of patients withdrew for this reason.^3^ The high dropout rate has placed a significant burden on the health care system. Although pain has been the nocebo symptom most studied thus far, accumulating evidence has revealed the powerful influence of nocebo on nausea and vomiting.^4^, ^5^ When exposed to a previous chemotherapy environment, approximately two out of every five cancer patients experienced nausea.^6^ It is critical to develop effective methods to weaken nocebo nausea.

Much effort has been made in recent years to elucidate the mechanisms underpinning nocebo effects. Several critical neurobiological pathways, including the activated hypothalamic–pituitary–adrenal (HPA) axis and cholecystokinin (CCK) systems, have been discovered.^2^, ^7^ However, the vast majority of studies focused on nocebo hyperalgesia, and knowledge about nocebo nausea is limited. One important reason is the particular scarcity of reliable animal models. It is well‐recognized that studying molecular pathways in people is tough. Inducing nocebo events in humans, on the other hand, is against healthcare ethics. It is therefore critical to develop a related animal model.

Although rats cannot truly vomit, they have been demonstrated to feel nausea and mimic vomiting behaviors.^8^, ^9^ In fact, early attempts to develop a nocebo‐related nausea animal model were effective in establishing a conditioned taste aversion paradigm.^10^, ^11^ In this paradigm, a Pavlovian conditioning procedure, which paired a novel flavor with an emetic drug (LiCl), was used to induce anticipatory nausea and taste avoidance in rats.^9^ Related molecular mechanisms were revealed to involve 5‐hydroxytryptamine (5‐HT), cannabinoid, and dopamine receptor systems.^12^ Conditioning alone, however, is not representative due to the lack of other nocebo mechanisms. Furthermore, rats do not effectively imitate the true nocebo process of clinical patients given a lack of treatment‐matched intervention. Taken together, establishing a nocebo nausea animal model by superimposing different mechanisms is promising.

Current evidence has identified the learning ability of rodents to experience unpleasant emotions in others (e.g., pain and fear).^13^, ^14^ However, no studies have been conducted to determine whether nausea can be acquired in rodents from social peers. Furthermore, our previous study found that migraine patients experienced strong nocebo nausea after taking placebos.^4^ The nitroglycerin (NTG) rodent model has been recognized as a reliable migraine model and is widely used in basic neuroscience research.^15^, ^16^ To better represent a clinical setting, the current study employed a contextual mode that matched migraine and treatment. Thus, the goals of this study were to (1) determine whether observing peers' nausea can elicit social nausea transfer in rats, (2) develop a new experimental rat model of nocebo nausea by combining observational learning with conditioning procedures, and (3) investigate the molecular mechanisms underlying nocebo nausea and whether these neurochemical alterations differed between the two inducing mechanisms.

## 2 MATERIALS AND METHODS

### 2.1 Animals

A total of 89 adult male Sprague–Dawley rats weighing 200–250 g were purchased from the Laboratory Animal Centre of Renmin Hospital of Wuhan University. The sample size was calculated based on the results of a previous study.^17^ All rats were kept in a specific pathogen‐free environment (temperature 22 ± 2°C, humidity 60 ± 5%, and 12‐h light/dark cycle). Before the experiment, rats were acclimated to the environment for 7 days and had ad libitum access to water and food. All experimental procedures were in accordance with animal welfare guidelines and approved by the Ethical and Animal Welfare Committees of Wuhan University.

### 2.2 Drug administration

To induce migraine attacks, rats were given an intraperitoneal (i.p.) injection of 10 mg/kg NTG every 3 days until the end of the trial. NTG was prepared from a stock solution of 5 mg/mL NTG, containing 30% propylene glycol, 30% alcohol, and water (Beijing Yimin, China), which was further diluted in 0.9% saline to a dose of 1 mg/mL.^18^ Sumatriptan (SUMA) at 0.6 mg/kg was administered subcutaneously (s.c.) 75 min after NTG injection to alleviate headaches.^19^, ^20^ A solution of 0.15 M LiCl was prepared with 0.9% saline and administered at a volume of 20 mL/kg, i.p.

### 2.3 Apparatus

The taste reactivity chamber consisted of a clear Plexiglas box (40 × 30 × 20 cm) with a shade cloth. The box was divided into two compartments by a white partition. Half of it was placed on a table, exposing the clear bottom of the other half. A mirror at a 45° angle was placed under the chamber to allow for viewing of the rat's ventral surface and orofacial responses. Meanwhile, behaviors were videotaped via a video camera.

### 2.4 Behavioral assays

#### 2.4.1 Von‐Frey test

To validate the migraine model, we performed a Von‐Frey test on rats using the Dixon up‐down method before the onset of the experiment and 2 h after NTG injection.^21^, ^22^ Briefly, the calibrated von Frey filament was put perpendicularly to the faces of rats, bending for 5 s under pressure. An active withdrawal of the head from the stimulus was viewed as a positive response. Five repeated measurements were taken, and the mechanical threshold was recorded if at least three of the measurements were positive. If no responses are observed, the filament size should be increased further.

#### 2.4.2 Nausea‐related behaviors

LiCl‐induced nausea behaviors consisted of lying‐on‐belly (LOB), reduced grooming, chin‐rubbing, head shaking, and oral responses (including tongue protrusions, mouth movements, and gaping).^17^, ^23^ LOB was defined as lying prostrate on the belly with a loss of body tension. Chin‐rubbing was defined as lowering the head to make the mouth advance and directly touch the floor or wall, and then retracting. Gaping, as a specific behavior similar to the vomiting of humans, referred to the rat's lowering the jawbone and exposing its lower teeth.

### 2.5 Procedures

Before starting the experiment, rats were deprived of water overnight for 5 days (5:00 p.m.–8:00 a.m.). In each rat, solution consumption was monitored daily by measuring the difference between its weight before and after 30 min of drinking water. Rats were placed in the taste reactivity chamber for 10 min every day for 3 days before the experiment to habituate to the surroundings. A computer‐generated random number was used to randomly allocate the animals to each group: 4 in the saline group, 4 in the NTG group, and 81 in the NTG‐SUMA group. The NTG‐SUMA animals were then randomly divided into six groups: 15 in the control (CON) group, 12 in the bystander (BST) group, 12 in the demonstrator 1 group, 12 in the conditioning (CDT) group, 15 in the BST + CDT group, and 15 in the demonstrator 2 group. All of these animals took part in Experiment 1. For the BST and BST + CDT groups, they were co‐housed with their demonstrators (demonstrator 1 and demonstrator 2), respectively, two per cage. The remaining groups were housed one per cage.

#### 2.5.1 Experiment 1

Experiment 1 was designed to investigate the role of different training paradigms in inducing nocebo nausea and the potential molecular mechanisms. Before that, we introduced a migraine‐treatment model to mimic the clinical context of patients. Specifically, rats in the NTG and NTG‐SUMA groups were administered NTG (i.p.) to trigger headaches. The saline group received a mix of 0.9% saline, propylene glycol, and alcohol. Then, rats were allowed to drink 0.1% sodium saccharin for 30 min to imitate the drug‐taking signal in a clinical setting. Followingly, rats in the NTG‐SUMA group were administered SUMA (s.c.) to alleviate headaches and given a nausea inducer to mimic a side effect of drugs. The saline and NTG groups received 0.9% saline (s.c.). The same procedure was performed every 3 days and given a total of five times.

##### Observational learning paradigm

**FIGURE 1:**
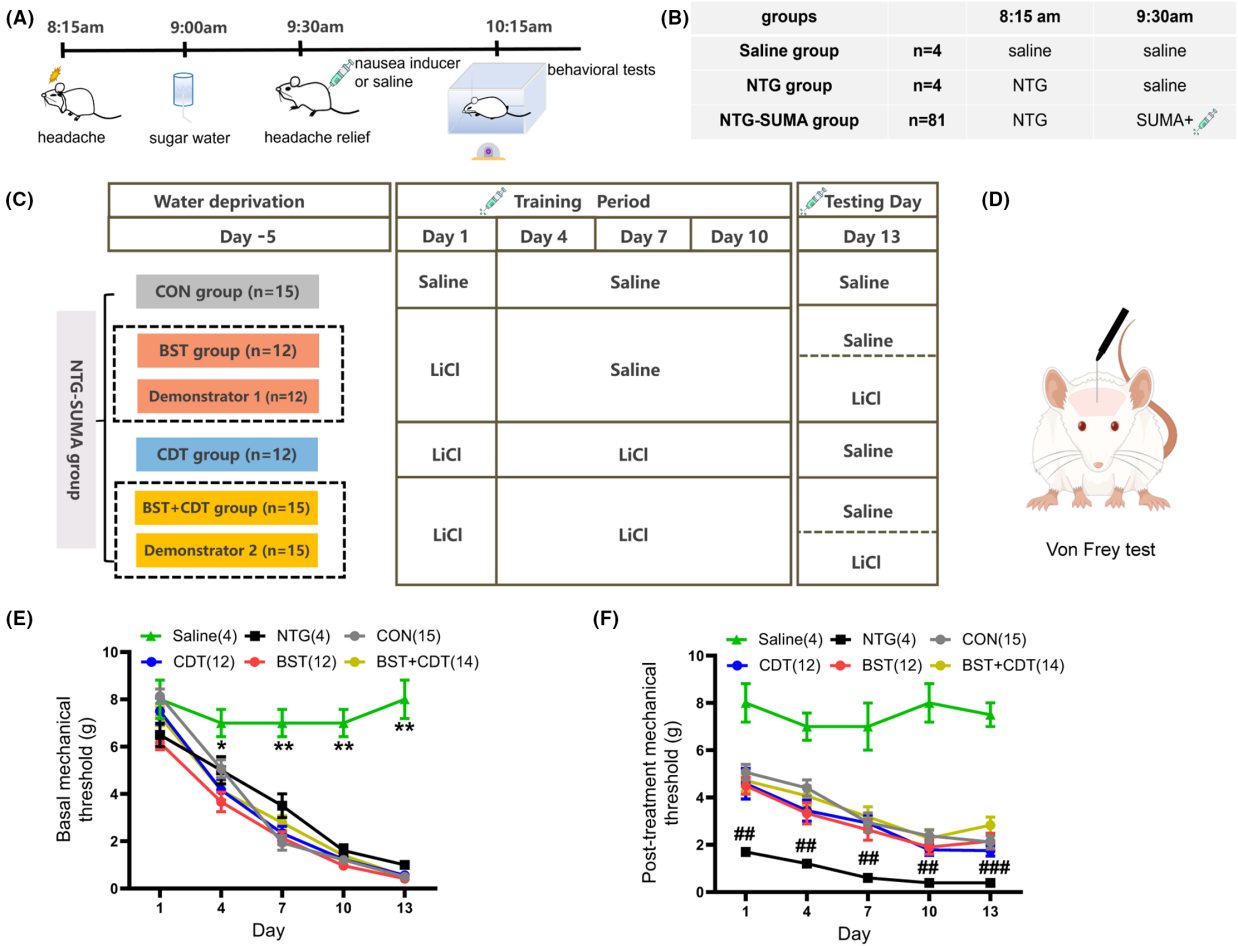
Experimental protocol for the establishment of a nocebo nausea model with different mechanisms. (A, B) The detailed time schedule of the training procedure for inducing migraine attacks and headache relief. (C) A schematic timeline diagram of administration plans for NTG‐SUMA rats in different nocebo mechanisms groups at 9:30 am, as a supplement to B. (D) A schematic draw of mechanical threshold measurements in Von‐Frey test. (E) Time course of basal mechanical threshold prior to NTG administration; two‐way ANOVA, *F* (5, 55) = 31.05, *p* < 0.001; Dunnett's multiple comparisons test, *p < 0.05; **p < 0.01, the saline group vs. all other groups. (F) Time course of post‐treatment mechanical threshold after SUMA administration; two‐way ANOVA, *F* (5, 55) = 23.4, *p* < 0.0001; Dunnett's multiple comparisons test, ^##^ *p* < 0.01, ^###^ *p* < 0.001, the NTG group vs. all other groups. Data are presented as mean ± SEM. ANOVA, analysis of variance; BST, bystander; CDT, conditioning; CON, control; NTG, nitroglycerin; SUMA, sumatriptan.

The observational learning paradigm follows other observational patterns,^24^ and familiar cagemates, not strangers, were considered optimal demonstrators. The BST rat was co‐housed with its demonstrator as described previously to increase familiarity. The first training day was set as a nausea‐experienced period for rats. The BST rat and its demonstrator received LiCl injections immediately following SUMA. Next, rats were placed in a taste reactivity chamber for 10 min of video recording, immediately returned to their cages, and recording continued for an additional 20 min. Over the next three training trials, LiCl was replaced with 0.9% saline to eliminate the impact of the first LiCl injection. On the test day (the 13th day of the experiment), the BST rat received 0.9% saline, and the demonstrator rat received a LiCl injection following SUMA. Both were put into the taste reactivity chamber to allow the BST rat to observe the peer's behaviors. Nausea‐related behaviors and the mechanical threshold were recorded (Figure 1C).

##### Conditioning paradigm

The conditioning paradigm was performed based on previous literature and consisted of 4 days, each spaced 72 h apart.^17^ On each training day, CDT rats were given LiCl injections following SUMA. Each rat then entered the taste reactivity chamber for 10 min. On the conditioned nausea day, each rat received i.p. 0.9% saline (a placebo) and was re‐exposed to the taste reactivity chamber. Animals' behaviors and the mechanical threshold were recorded as above.

The CON rat was given 0.9% saline following SUMA on both training and test days. The BST + CDT rat was exposed to both observational learning and conditioning paradigms simultaneously. Two observers blinded to group allocations recorded the video. Another pair of observers who were not aware of the experimental protocol watched video and documented behavioral data. Experiment 2 was continued with the twenty‐three rats, and additional rats were sacrificed after completing behavioral tests.

#### 2.5.2 Experiment 2

Experiment 2 aimed to assess the retention time of nocebo nausea responses under different mechanisms. This experiment included a total of 23 rats (*n* = 6 in the CON, BST, and CDT groups, and *n* = 5 in the CON+BST group). To investigate how nocebo effects changed in the short term, animals were observed for behaviors 60, 120, and 180 min after evoking nocebo nausea. The paradigm used to examine long‐term behavior changes included 4 days separated by 72 h. Three days after the test day, rats were administered NTG to induce headaches and then treated as described above. After that, rats were given 0.9% saline i.p. (as a placebo) and placed into the taste reactivity chamber. The mechanical threshold, the consumption volume of 0.1% sodium saccharin, and nausea behaviors were all recorded. The behaviors were elicited at 3‐day intervals until the 25th day of the experiment. All rats were sacrificed at the end of the experiment.

### 2.6 Immunofluorescence assay

Brain tissues were fixed in 4% paraformaldehyde, embedded in paraffin, and cut into coronal slices of 5 μm. Repair antigens were performed following deparaffinization and rehydration. The sections were then permeabilized using 0.3% Triton X‐100 and washed with PBS 3 times. After blocking with 5% bovine serum albumin (BSA), the sections were incubated with mouse anti‐c‐Fos (1:200, Proteintech) and rabbit anti‐CCK (1:100, Ablepsience, Henan, China) at 4°C overnight. Cy3‐labeled goat anti‐mouse IgG and FITC‐labeled goat anti‐rabbit IgG (both 1:200, Servicebio) were used as secondary antibodies. The sections were washed in PBS 3 times and then stained with DAPI (Servicebio) to reveal nuclei. At least 3 representative, non‐overlapping images at 400× magnification were obtained with a fluorescence microscope (Olympus BX51). The immunoreactivity levels were determined by analyzing the positive stained area with Image J software.

### 2.7 Real‐Time PCR (qPCR)

Total RNAs were freshly extracted from the medulla of rats using TRIzol (Servicebio). A NanDrop ND1000 Spectrometer was used to measure the RNA concentration in each sample. Equal amounts of RNA (2 μg) were reverse transcribed into cDNA using the SweScript All‐in‐One First‐Strand cDNA Synthesis SuperMix kit (G3337‐100, Servicebio). QPCR was performed in a 20 μL volume with SYBR Green (G3324‐15, Servicebio) under a Light Cycler 480 (Roche) system. The thermal cycle parameters were programmed according to the manufacturer's instructions. The sequences of primers used are provided in Table S1.

### 2.8 ELISA

On day 13 of the experiment, blood samples (1 mL, *n* = 6/group) were collected in 1% heparin sodium tubes before being sacrificed. For obtaining serum, samples were centrifuged at 3000 rpm at 4°C for 15 min. Concentrations of CCK, ACTH, and cortisol were measured with Quantikine ELISA kits (SP12450, SP13190, SP12609, Spbio) under the guidance of operation instructions. Bicinchonic acid (BCA) assay was used to quantify protein at 578 nm.

### 2.9 Western blot assay

Rat tissues in the medulla were freshly dissolved in RIPA buffer, which consisted of a protease inhibitor cocktail and phenyl‐methylsulfonyl fluoride (PMSF). In a 12.5% SDS‐PAGE gel, equal amounts of proteins (20 μg) were separated and electrophoretically transferred onto a polyvinylidene difluoride (PVDF) membrane. After blocking with 5% nonfat dry milk, the membranes were incubated with mouse anti‐c‐Fos (1:2000, Proteintech) and rabbit anti‐GAPDH (1:3000, Servicebio) at 4°C overnight. The following morning, the membranes were washed using TBST and then incubated with HRP‐labeled goat anti‐mouse and HRP‐labeled goat anti‐rabbit (both 1:5000, Servicebio) for 1 h. The chemiluminescence system (ChemiDocTM XRS+, BioRad) was used to visualize protein blotting. Protein expression was quantified using Image J software 1.8.0.

### 2.10 Statistics analysis

Data analyses were carried out using SPSS 26.0 and the GraphPad Prism 8.0 version. The Shapiro–Wilk test and normal Q–Q plots were used to check data normality. Normally distributed data are presented as the mean ± standard error (SEM). Differences between groups were analyzed using Student's unpaired two‐tailed *t*‐test or one‐way or two‐way analysis of variance (ANOVA). Pairwise comparison was performed by Bonferroni or Dunnett post‐hoc tests. Data from Skewed distributions are represented as medians with an interquartile range (IQR). Statistical analyses were conducted using the non‐parametric Kruskal‐Wallis *H* test or Scheirer‐Ray‐Hare test, with Bonferroni correction for multiple‐group comparisons. Statistical significance is considered at *p* < 0.05.

## 3 RESULTS

### 3.1 SUMA relieved acute hyperalgesia in NTG‐induced migraine model

The majority of rats gained weight normally and consumed at least 8 mL of water within 30 min after the water deprivation training. However, one BST + CDT rat who planned to join Experiment 2 had severe weight loss and was thus excluded. To verify the successful establishment of the migraine model, the mechanical threshold was measured. As shown in Figure 1E, the basal mechanical threshold of rats receiving repeated NTG injections gradually decreased and was significantly different from the saline group (two‐way ANOVA, *p* < 0.001). To investigate the efficacy of SUMA in treating migraine, we assessed the mechanical threshold 45 min after SUMA injection. The results showed that SUMA administrations significantly relieved acute hyperalgesia in migraine rats on all experiment days (Figure 1F, two‐way ANOVA, *p* < 0.001).

### 3.2 Observing peers experiencing nausea partly elicited nausea in BST rats

**FIGURE 2:**
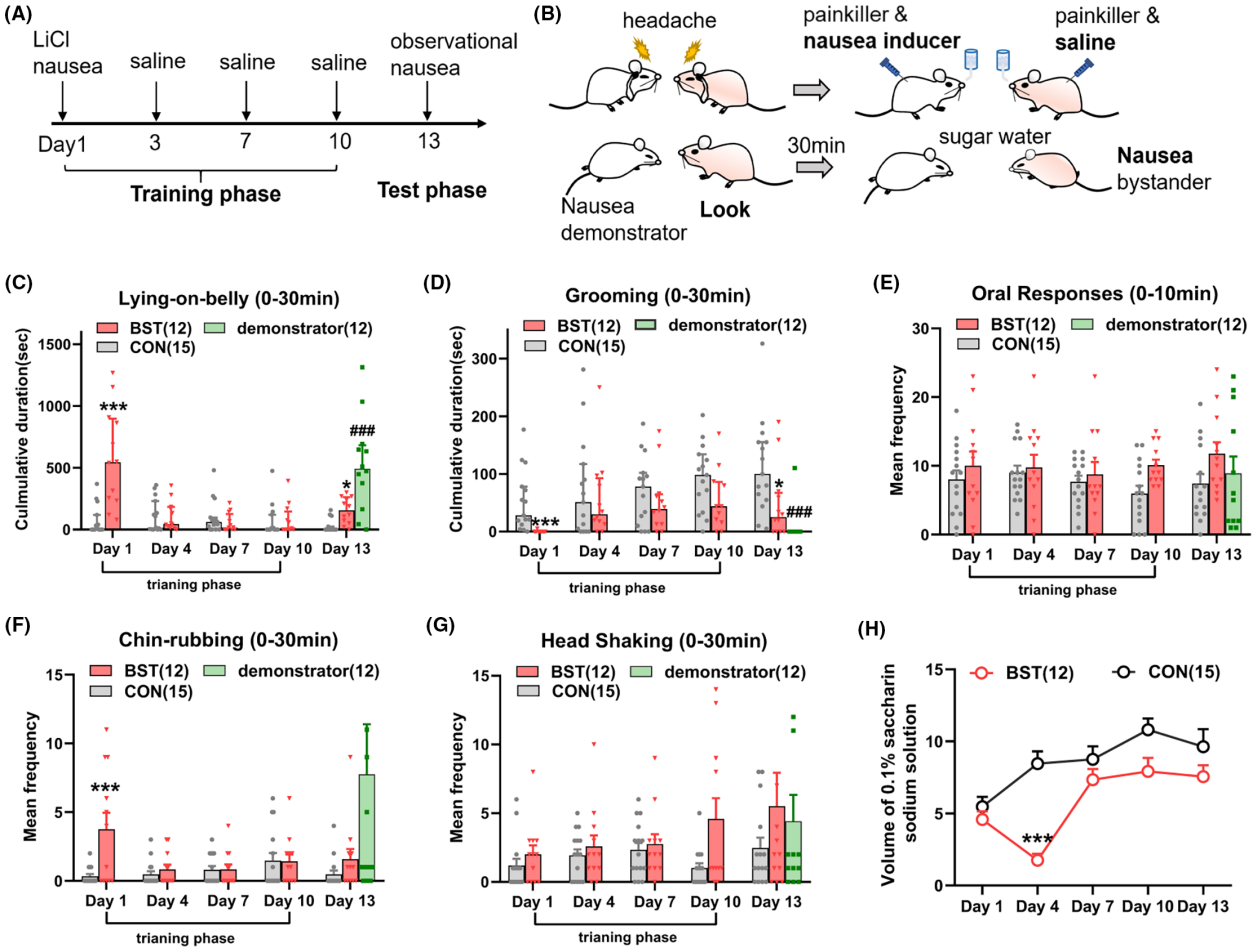
The observational learning partly elicited nocebo nausea in migraine rats. (A) Schedule of observational learning training in BST rats. (B) Cartoon illustrating a BST rat looking at a nausea demonstrator on the test phase. (C, D) Mean duration of Lying‐on‐belly and mean duration of grooming on the training and test phase in the CON and BST rat; (Bars indicate medians with IQR; (C) Scheirer–Ray–Hare test, *H* (1) = 12.184, *p* < 0.001, and (D) *H* (1) = 13.671, *p* < 0.001). (E–G) Mean frequency of oral responses, chin‐rubbing, and head shaking on the training and test phase in the CON and BST rat (bars indicate mean with SEM; (F) two‐way ANOVA, *F* (1, 25) = 4.251, *p* = 0.049). (H) The saccharin consumption within 30 min on the training and test phase in the CON and BST rat; two‐way ANOVA, *F* (1, 25) = 12.45, *p* = 0.002. *p < 0.05; **p < 0.01, ***p < 0.001 (CON vs. BST), ^###^ *p* < 0.001 (CON vs. demonstrator). ANOVA, analysis of variance; BST, bystander; CON, control.

We provided a nausea peer to a normal rat (a BST rat) to elicit the observational nausea. Before that, all BST rats were allowed to pre‐experience nausea, which was considered to be able to provoke stronger observational effects.^14^, ^25^ Compared with the CON rat, the BST rat after LiCl injection on day 1 demonstrated more nausea‐related behaviors, including LOB (Figure 2C, CON vs. BST, *p* < 0.001), reduced grooming (Figure 2D, *p* < 0.001) and chin‐rubbing (Figure 2F, *p* < 0.001). There was a clear taste avoidance of 0.1% saccharin solution in BST rats on the second training (Figure 2H, *p* < 0.001).

To avoid conditioned memory formation, LiCl was replaced with 0.9% saline during the following three training sessions. On the test day, BST rats did not show a significant decrease in saccharin solution consumption, suggesting that there was no nausea anticipation. We next examined the role of a brief (30 min), direct social nausea observation in the BST rat. After 30 min of observation, the BST rat receiving 0.9% saline exhibited a more significant LOB response (Figure 2C, CON vs. BST, *p* = 0.031) and reduced grooming (Figure 2D, *p* = 0.032).

### 3.3 Conditioning with repeated LiCl injection elicited anticipatory nausea in CDT rats

**FIGURE 3:**
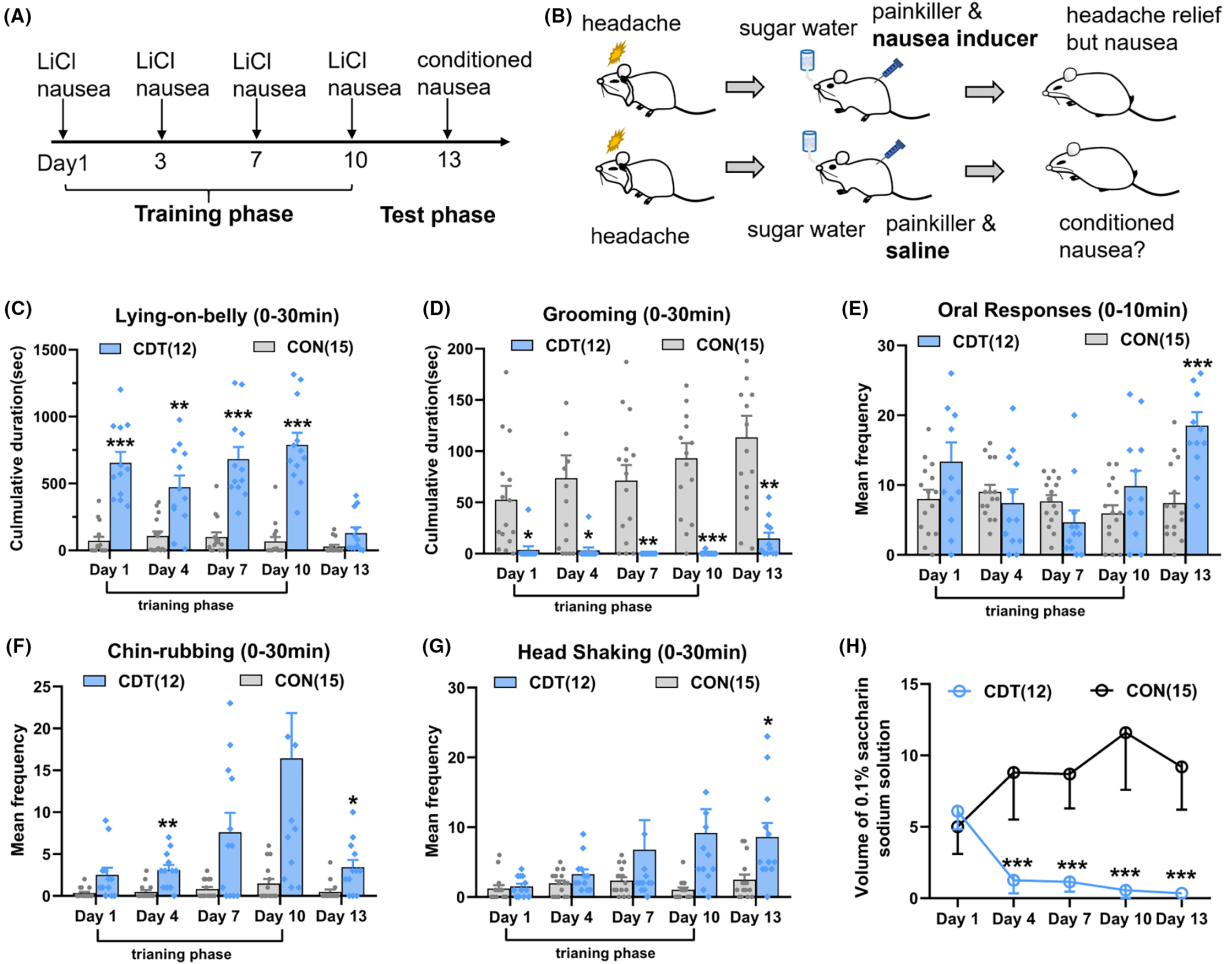
The Pavlovian conditioning elicited nocebo nausea in migraine rats. (A) Schedule of conditioning training in CDT rats. (B) Cartoon illustrating a CDT rat acquiring headache relief and nausea inducer simultaneously on the training phase, and only acquiring headache relief on the test phase. (C, D) Mean duration of Lying‐on‐belly and mean duration of grooming on the training and test phase in the CON and CDT rat (bars indicate mean, with SEM; (C) two‐way ANOVA, *F* (1, 25) = 105.4, *p* < 0.001, and (D) *F* (1, 25) = 26.01, *p* < 0.001). (E–G) Mean frequency of oral responses, chin‐rubbing, and head shaking on the training and test phase in the CON and CDT rat (bars indicate mean, with SEM; (E) two‐way ANOVA, *F* (1, 25) = 4.104, *p* = 0.046, (F) *F* (1, 25) = 23.5, *p* < 0.001, and (G) *F* (1, 25) = 10.11, *p* = 0.004). (H) The saccharin consumption within 30 min on the training and test phase in the CON and CDT rat; Scheirer‐Ray‐Hare test, *H* (1) = 71.125, *p* < 0.001. *p < 0.05; **p < 0.01, ***p < 0.001. ANOVA, analysis of variance; CDT, conditioning; CON, control.

We examined the conditioning effects of repeated LiCl and saccharin solutions on nocebo nausea. A single LiCl injection on each training day was enough to produce noticeable nausea behaviors in CDT rats (Figure 3C,D,F). Following four training sessions, CDT rats ingested much less 0.1% saccharin solution than CON rats, demonstrating that a negative expectation had been generated by associating the flavor stimulus to nausea induction (Figure 3H, Scheirer‐Ray‐Hare test, *p* < 0.001). On the test day, CDT rats given saline injections had considerably less grooming time (Figure 3D, CON vs. CDT, *p* = 0.002) and more oral responses (Figure 3E, *p* < 0.001), chin‐rubbing (Figure 3F, *p* = 0.037), and head shaking (Figure 3G, *p* = 0.014). These findings indicated that the conditioning paradigm could elicit strong nocebo nausea responses.

### 3.4 Superposition of the two mechanisms led to a further decrease in grooming time

**FIGURE 4:**
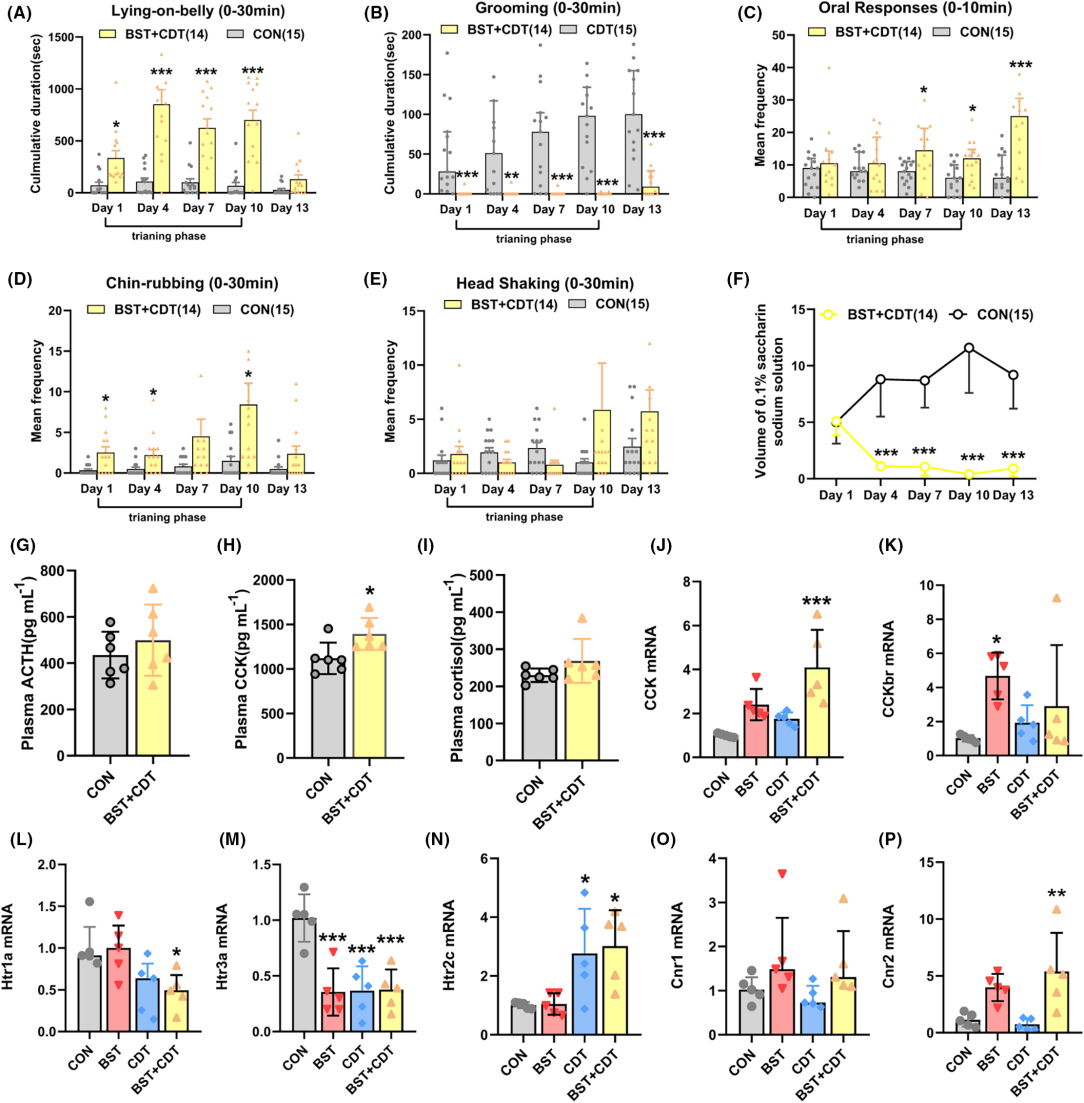
Double training paradigms induce nocebo nausea behaviors and involve activation of the CCK and 5‐HT systems. (A, B) Mean duration of Lying‐on‐belly and mean duration of grooming on the training and test phase in the BST + CDT rat ((A) bars indicate mean with SEM, two‐way ANOVA, *F* (1, 27) = 71.22, *p* < 0.001, and (B) bars indicate medians with IQR, Scheirer‐Ray‐Hare test, *H* (1) = 77.186, *p* < 0.001). (C–E) Mean frequency of oral responses, chin‐rubbing, and head shaking on the training and test phase in the BST + CDT rat ((C) bars indicate medians with IQR, Scheirer‐Ray‐Hare test, *H* (1) = 19.919, *p* < 0.001, and (D) bars indicate mean with SEM, two‐way ANOVA, *F* (1, 27) = 9.029, *p* = 0.006). (F) The saccharin consumption within 30 min on the training and test phase in the BST + CDT rat; Scheirer‐Ray‐Hare test, *H* (1) = 85.136, *p* < 0.001. (G–I) Mean serum concentrations of cortisol, ACTH, and CCK in the CON and BST + CDT rat; *n* = 6/group; ((H) bars indicate mean with SEM, unpaired *t*‐test, *t* = 6.662, df = 10, *p* = 0.024). (J–P) The RNA expressions of key molecules involved in the nocebo nausea in the medulla. *n* = 5/group ((J) bars indicate mean with SEM, one‐way ANOVA, *F* (3, 16) = 9.9, *p* = 0.001, (K) *F* (3, 16) = 11.55, *p* < 0.001, (M) *F* (3, 16) = 12.43, *p* = 0.001, (N) *F* (3, 16) = 5.916, *p* = 0.007, (P) *F* (3, 16) = 7.392, *p* = 0.003, and (L) bars indicate medians with IQR, Kruskal‐Wallis *H* test, *H* (3) = 9.834, *p* = 0.020). *p < 0.05, **p < 0.01, ***p < 0.001 vs. the CON group; 5‐HT, 5‐hydroxytryptamine; ACTH, adrenocorticotropic hormone; ANOVA, analysis of variance; BST, bystander; CCK, cholecystokinin; CCKbr, Cholecystokinin B Receptor; CDT, conditioning; cnr, cannabinoid receptor; CON, control; htr, 5‐hydroxytryptamine receptor.

To investigate if stronger nocebo nausea would be evoked under the two mechanisms, we used the observational learning and conditioning paradigms to train rats together. Two‐factor Scheirer‐Ray‐Hare analysis showed that compared with CON rats, BST + CDT rats demonstrated less grooming time (Figure 4B, *p* < 0.001) and more oral responses (Figure 4C, *p* < 0.001). An increased tendency towards LOB, chin‐rubbing, and head shaking was also observed, although there were no statistical differences (Figure 4A,D,E). NOC rats, like CDT rats, exhibited obvious conditioned taste avoidance (Figure 4F, Scheirer–Ray–Hare test, *p* < 0.001).

When performing multiple‐group comparisons, BST + CDT rats appeared to have a further response to decreasing grooming time (Figure S1B, Kruskal–Wallis H test, *p* < 0.001) and increasing oral responses (Figure S1C, one‐way ANOVA, *p* < 0.001), compared with single mechanism groups. Generalized estimating equations were used to further probe the interaction effects between observational learning and conditioning. The results showed there were significant main effects both of observational learning (*p* = 0.019) and conditioning (*p* < 0.001) in reducing grooming time, and a significant interaction between them (*p* = 0.021). These findings suggested that the superposition of two mechanisms could cause a further enhancement of nocebo nausea responses, at least partly.

### 3.5 CCK, 5‐HT, and cannabinoid systems were activated in nocebo nausea

Given the hyperactivity of CCK and the HPA axis in other nocebo symptoms (e.g., pain),^26^ we explored the roles of these substances in nocebo nausea. The serological results of ELISA tests demonstrated that, compared to CON rats, peripheral CCK release increased (Figure 4H, unpaired *t*‐test, *p* = 0.024) but not cortisol or ACTH (Figure 4G,I) in BST + CDT rats. To investigate if CCK played roles in the central nervous system, we further measured the mRNA expression of CCK and its receptors in the medulla, where the emetic area was located. Likewise, we found a significantly increased expression of CCK mRNA in the medulla of BST + CDT rats (Figure 4J, one‐way ANOVA, *p* = 0.001). The CCKB receptor mRNA was up‐regulated (Figure 4K, *p* < 0.001), meaning that the CCKB receptor but not the CCKA receptor might mediate the modulatory actions of CCK (Figure S1F).

Furthermore, we assessed the mRNA levels of nausea signaling‐related molecules in the medulla to investigate the potential signal pathway. The results revealed significant changes with 5‐HT systems, including decreased mRNA levels of 5‐HT 1A (Figure 4L, Kruskal‐Wallis H test, *p* = 0.020) and 5‐HT 3A receptors (Figure 4M, one‐way ANOVA, *p* = 0.001), as well as increased mRNA levels of the 5‐HT 2C receptor (Figure 4N, *p* = 0.007). Particularly, we also observed changes in the mRNA levels of 5‐HT 1A and 5‐HT 2C receptors in BST + CDT rats that were consistent with those in CDT rats rather than in BST rats. This finding meant that these alterations in 5‐HT receptors might be unique to conditioned nausea. An increased mRNA level of cannabinoid receptor 2 (cnr2) was also found in BST + CDT rats (Figure 4P, one‐way ANOVA, *p* = 0.003), and a similar change appeared in BST rats, although the difference was not statistically significant. We found no changes in mRNA levels for dopamine receptor systems (Figure S1G,H). The above results indicated that CCK, 5‐HT, and cannabinoid systems were potentially involved in the development of nocebo nausea.

To further determine whether the alterations were caused by nocebo effects or by LiCl itself, we examined the levels of relevant genes in the medulla of rats without LiCl and rats 72 h after LiCl injections. CCK mRNA levels were shown to be high in rats 72 h after LiCl injections (Figure S2A, unpaired *t*‐test, *p* = 0.006). However, no alterations in the 5‐HT and cannabinoid systems were observed (Figure S2).

### 3.6 Brain activation maps in nocebo nausea rats

**FIGURE 5:**
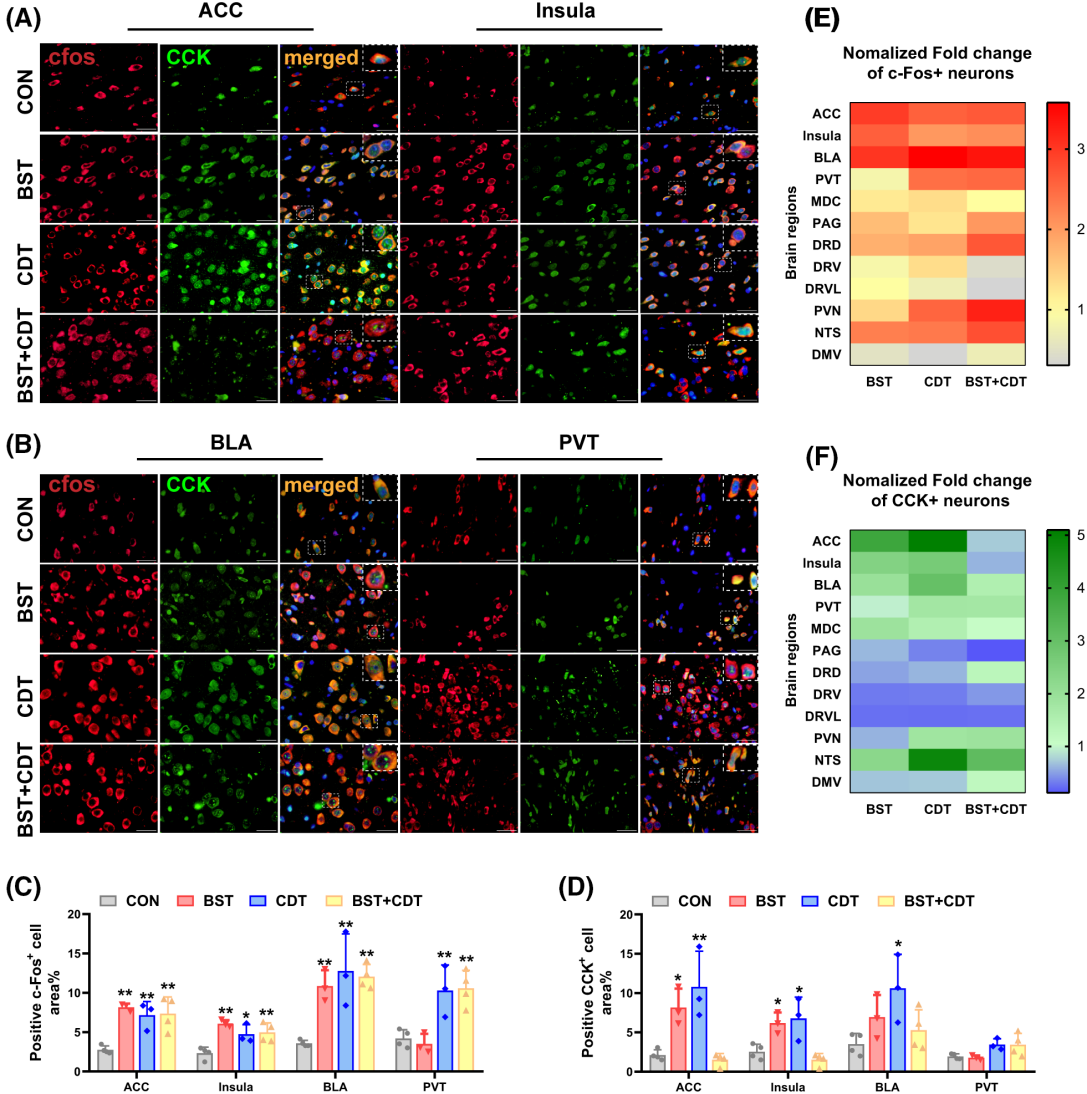
Nocebo nausea activated c‐Fos and CCK neurons in brain regions related to cognition and emotion. (A) Representative immunofluorescence staining showing increased positive c‐Fos (red) and CCK (green) neurons in ACC and insula. The nuclear translocation of CCK in ACC increased in the CDT and BST + CDT rats. Yellow and orange colors represent the colocalization of c‐Fos and CCK. The white box is an enlarged region. Scale bar = 20 μm. (B) Representative immunofluorescence staining showing increased positive c‐Fos (red) neurons in BLA and PVT. The nuclear translocation of CCK (green) in BLA increased in the BST and BST + CDT rat. Scale bar = 20 μm. (C) Quantification analysis of the area of positive c‐Fos neurons in the ACC, insula, BLA, and PVT; *n* = 3–4/group, one‐way ANOVA, *F*_ACC_ (3, 10) = 10.76, *p* = 0.002, *F*_insula_ (3, 10) = 9.581, *p* = 0.003, *F*_BLA_ (3, 10) = 11.27, *p* = 0.002, and *F*_PVT_ (3, 10) = 11.53, *p* = 0.001. (D) Quantification analysis of the area of positive CCK neurons in the ACC, insula, BLA, and PVT; *n* = 3–4/group, one‐way ANOVA, *F*_ACC_ (3, 10) = 12.58, *p* = 0.001, *F*_insula_ (3, 10) = 10.28, *p* = 0.002, and *F*_BLA_ (3, 10) = 3.908, *p* = 0.044. (E) A heat map comparing the normalized fold change in the area of positive c‐Fos neurons in different brain regions. (F) A heat map comparing the normalized fold change in the area of positive CCK neurons in different brain regions. Data are presented as mean ± SEM; *p < 0.05, **p < 0.01, ***p < 0.001 vs. the CON group; ACC, anterior cingulate; ANOVA, analysis of variance; BLA, basalateral amygdala; BST, bystander; CCK, cholecystokinin; CDT, conditioning; CON, control; DMV, dorsal motor nucleus of the vagus; DRD, dorsal raphe nucleus‐dorsal part; DRV: dorsal raphe nucleus‐ventral part; DRVL: dorsal raphe nucleus‐ventrolateral part; MDC, mediodorsal thalamus‐central subnucleus; NTS, nucleus tractus solitarius; PAG, periaqueductal gray; PVN, hypothalamic paraventricular nucleus; PVT, thalamic paraventricular nucleus.

To determine which brain regions participated in nocebo nausea and what role CCK played, we used immunofluorescence to map c‐Fos and CCK activations in brain. A significant increase of c‐Fos positive neurons was detected in brain regions related to cognition and emotion, such as the anterior cingulate (ACC), insula, and basalateral amygdala (BLA) (Figure 5A–C, one‐way ANOVA, *p*_ACC_ = 0.002, *p*_insula_ = 0.003, and *p*_BLA_ = 0.002). The co‐localization analysis revealed an increase in nuclear CCK in CDT and BST + CDT rats in ACC, which exhibited a high degree of co‐localization with c‐Fos‐positive neurons (Figure 5A). In BLA, BST, and BST + CDT rats displayed elevated nuclear CCK levels, whereas CCK in CON rats was primarily localized in the cytoplasm (Figure 5B). No significant changes in the suborganelle localization of CCK were observed in the hippocampus or mediodorsal thalamus‐central part (MDC) (Figures S3 and S4). These findings suggested that the differential distribution of CCK in the nuclei of various brain regions may play a crucial role in driving nocebo effects.

Furthermore, we investigated the role of periaqueductal gray (PAG) and the dorsal raphe nucleus (DRN), a gathering site of 5‐HT in the brain, which contained the dorsal part (DRD), ventral part (DRV), ventrolateral part (DRVL). One‐way ANOVA analysis showed increased activation of c‐Fos neurons in PAG and DRD (Figure S5B,F, one‐way ANOVA, *p*_PAG_ <0.001, and *p*_DRD_ = 0.006), with decreased c‐Fos expressions in DRVL (Figure S4B,F, *p* < 0.001). Different from c‐Fos, CCK expressions were obviously decreased in PAG, DRVL, and DRV (Figures S4 and S5, one‐way ANOVA, *p*_DRVL_ < 0.001, *p*_DRV_ = 0.014, and *p*_PAG_ < 0.001). We also found that both c‐Fos and CCK were up‐regulated in the hypothalamic paraventricular nucleus (PVN), and thalamic paraventricular nucleus (PVT), particularly in CDT and BST + CDT rats (Figure 5C, Figure S5E,G, one‐way ANOVA, c‐Fos: *p*_PVN_ = 0.003, and *p*_PVT_ = 0.001; CCK: *p*_PVN_ = 0.005). Nucleus tractus solitarius (NTS) was also significantly activated (Figure S5E–G, c‐Fos: *p* = 0.022; CCK: *p* = 0.001), while there was no change of c‐Fos and CCK expressions in the dorsal motor nucleus of the vagus (DMV) (Figure S4E–G).

We further assessed the impact of LiCl injection itself on brain activations. The results demonstrated that, when compared to no LiCl delivery, ACC and BLA exhibited more positive c‐Fos neurons 72 h after LiCl injections (Figure S6A,B, unpaired *t*‐test, *p*_ACC_ = 0.005, and *p*_BLA_ = 0.032). CCK expression was likewise observed to be considerably lower in the PAG and higher in the NTS 72 h after LiCl injection (Figure S6C, unpaired *t*‐test, *p*_PAG_ = 0.004, and *p*_NTS_ = 0.002). Further analysis revealed a large increase in c‐Fos activations in nocebo trainings than plain LiCl injections, implying that nocebo effects may still play a role in these variations. Changes in CCK in the PAG and NTS, however, should be explained carefully.

### 3.7 The nocebo nausea in rats could be preserved for at least 12 days

**FIGURE 6:**
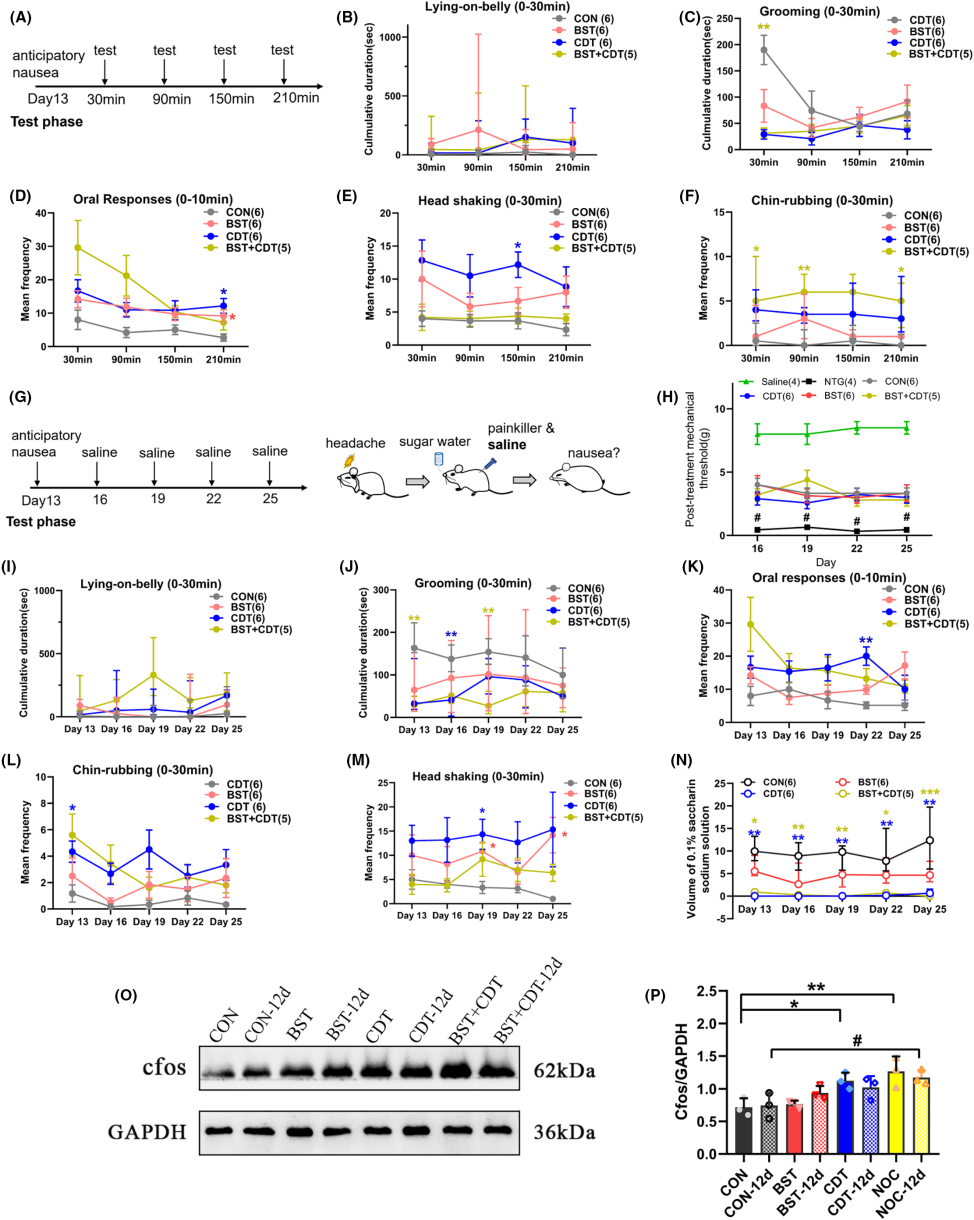
Time course of nocebo nausea responses after the first induction. (A) Schedule of behavioral test within 4 h after the first nocebo nausea induction. (B–F) The nocebo‐induced nausea behaviors faded over time after the first induction. Oral responses could be sustained for at least 210 min in both BST and CDT groups ((C) two‐way ANOVA, *F*_time effect_ (2, 40) = 3.028, *p* = 0.029, (D) *F*_group effect_ (3, 19) = 8.501, *p* < 0.001, *F*_time effect_ (2, 38) = 7.719, *p* = 0.001, (E) *F*_group effect_ (3, 19) = 3.336, *p* = 0.04, and (F) Scheirer‐Ray‐Hare test, *H*_group effect_ (3) = 34.973, *p* < 0.001). (G) Schedule of behavioral test within 12 days after the first nocebo nausea induction. (H) Post‐treatment mechanical threshold 45 min after SUMA administration within 12 days of first nocebo nausea induction; two‐way ANOVA, *F* (5, 25) =33.23, *p* < 0.001. (I–M) The change pattern of nausea behaviors within 12 days of the first nocebo nausea induction; ((J) Scheirer‐Ray‐Hare test, *H*_group effect_ (3) = 29.262, *p* < 0.001, (K) *F*_group effect_ (3, 19) = 8.270, *p* = 0.001, (L) *F*_group effect_ (3, 19) = 4.104, *p* = 0.021, *F*_time effect_ (3, 55) = 2.081, *p* = 0.039, and (M) *F*_group effect_ (3, 19) = 3.908, *p* = 0.025). (N) Time course of saccharin consumption within 12 days after the first nocebo nausea induction; Scheirer‐Ray‐Hare test, *H*_group effect_ (3) = 90.120, *p* < 0.001. (O) Western blotting of c‐Fos protein in the medulla on the test day and 12 days after the nocebo nausea induction. (P) Quantification analysis illustrating c‐Fos expressions increasing on the test day and remaining higher in the BST + CDT rat 12 days later; one‐way ANOVA, *F* (3, 8) = 9.584, *p* = 0.005 (on the test day), and *F* (3, 8) = 4.163, *p* = 0.047 (12 days after testing). *p < 0.05, **p < 0.01, ***p < 0.001 vs. the CON group; ^#^ *p* < 0.05 vs. the NTG group. ANOVA, analysis of variance; BST, bystander; CDT, conditioning; CON, control.

To investigate the retention period of nocebo nausea responses, we lengthened the first behavior observation to 4 h. As shown in Figure 6B–F, most of the nausea behaviors gradually subsided with time after the first induction. However, rats showed significantly higher oral responses (Figure 6D, two‐way ANOVA, *p* < 0.001) and chin‐rubbing behaviors (Figure 6F, Scheirer‐Ray‐Hare test, *p* < 0.001) at 210 min, indicating that nausea responses could be partly sustained for at least 210 min. We next examined whether nocebo nausea responses could be evoked again under the same condition. On day 12 after nocebo nausea evoking, higher nausea responses were observed, although there were no statistically significant differences (Figure 6I–M). Unexpectedly, BST rats also displayed higher head shaking 12 days after nausea induction (Figure 6M, two‐way ANOVA, *p* = 0.025). We also observed sustained aversion to saccharin solution in rats within 12 days, suggesting that the nausea anticipation was preserved (Figure 6N, Scheirer‐Ray‐Hare test, *p* < 0.001).

Furthermore, we detected protein levels of c‐Fos in the medulla on the test day and 12 days after nocebo nausea induction. Compared to CON rats, c‐Fos protein levels were obviously increased in CDT and CDT + BST rats on the test day (Figure 6O,P, CON vs. CDT: *p* = 0.027, CON vs. BST + CDT: *p* = 0.005), and remained higher in BST + CDT rats 12 days after nausea induction (Figure 6P, *p* = 0.025). No significant decrease of c‐Fos protein on the 12th day after nausea induction was found, compared with the test day. The above results demonstrated that in our rat models, parts of nocebo behaviors and the anticipation of nausea could be sustained at least for 12 days.

## 4 DISCUSSION

This study successfully established a nocebo‐nausea animal model with dual mechanisms of observational learning and conditioning. When two mechanisms were combined, nausea responses increased much more than when conditioning was applied alone, though this benefit was only evident in terms of reduced grooming time. Twelve days after the initial induction, these nausea behaviors still exhibited an increasing trend, and the expectation of nausea was consistently intense. The investigation of potential mechanisms for nocebo nausea revealed the critical importance of the CCK and 5‐HT systems. Brain activations related to cognition, memory, and affection have been shown to contribute to developing nocebo. Our experiments illustrated the difficulty and complexity of eliciting nocebo effects in animals. Nonetheless, this animal model has made great progress and has distinct advantages.

This is the first animal model to try to simulate an actual clinical situation involving nocebo occurrence. A contextual mode that matched disease and treatment was used in our model. To date, most of the knowledge about nocebo stems from the side effects reported by patients receiving placebos, which were viewed as nocebo responses.^27^ Previous animal models mostly focused on a conditioned taste aversion model based on a procedure for nausea conditioning with sweet taste stimuli.^10^, ^11^ It should be noted that a single conditioning mechanism is insufficient to account for nocebo effects. These models, on the other hand, are all based on healthy animals, possibly due to the difficulties of inducing nocebo effects in diseased animals.^28^ However, the fact is that nocebo effects are inextricably linked with illness states.

We also identified that, for the first time, the mere observation of peers' nausea could elicit social nausea transfer in rats. The social learning mechanism, which differs from conditioning, is thought to be a key mediator of expectations and nocebo effects.^29^ As in other unpleasant emotions (e.g., pain), our experiments demonstrated that rats have the ability to learn nausea from other rats.^13^ However, nausea responses obtained through social learning were considerably weaker than those obtained through conditioning. Indeed, there has been little research to compare the evoking strengths of the two mechanisms in nocebo effects. Only a recent study^30^ investigated the role of distinct intensity conditioning methods in nocebo, and the results showed that more conditioning training is associated with stronger nocebo hyperalgesia. Thus, the difference in training days between the two inducing mechanisms was a possible explanation for the above phenomenon. The combination of different expectation induction procedures is thought to elicit significantly stronger nocebo effects.^5^ We further made an attempt to combine observational learning with conditioning to evoke stronger nocebo nausea in rats. Just one behavioral indicator of nausea, however, was observed to induce stronger effects. It is unclear what complex interactional impact would be produced between the two inducing mechanisms. Still, the present animal model was considered to be more representative of the actual occurrence of nocebo.

Next, the underlying neurobiological mechanisms of nocebo nausea were examined. It has been proposed that anticipation anxiety and HPA axis activation are closely related to nocebo effects.^31^, ^32^ However, the HPA axis was not hyperactive in the current model. One crucial discovery was the considerable upregulation of serum CCK levels. As a physiological peptide produced from the intestine, CCK plays an important role in digestion and the control of appetite.^33^ Recent studies demonstrated that exogenous CCK at high dosages could directly trigger nausea in rodents,^34^, ^35^ and the process might be mediated by NTS that received gastrointestinal vagal afferent inputs.^33^ These findings identified the potential role of peripheral CCK in driving nocebo nausea and the possibility of serving as a novel serologic nocebo marker.

Additionally, a large body of research demonstrated that CCK functioned as a neuromodulator in the brain, controlling actions linked to feeding, mood, memory, and cognitive function.^36^, ^37^, ^38^ It has also been implicated as a crucial regulator of nocebo hyperalgesia.^31^, ^39^ Similarly, we discovered significantly increased CCK mRNA expressions in the medulla when nocebo nausea occurred, which further determined the unique contribution of CCK to nocebo effects. However, the same change in CCK gene expression was found in rats only receiving LiCl injections. Thus, the complete exclusion of the impact of pharmacological actions on CCK alterations is difficult. Another potential explanation is that increased CCK is the product of nocebo mimicking the pharmacological effect of LiCl. Prior research has proven the specific imitation ability of nocebo for drugs.^40^ A representative example was that side effects reported by patients receiving placebos relied on side effects reported by patients receiving active medications.^4^ We suggest future work is needed to further investigate the accurate source of CCK.

The signaling pathways connected to nausea, including the 5‐HT, cannabinoid, and dopamine receptor systems, were also investigated.^12^ The findings revealed considerable changes in 5‐HT receptor systems: the 5‐HT 2C receptor's mRNA expression had increased, while that of the 5‐HT 1A and 5‐HT 3A receptors had dropped. 5‐HT3 receptor antagonists have been shown in clinical trials to improve chemotherapy‐induced nausea and vomiting.^41^, ^42^ As an ion channel, the 5‐HT3 receptor mediates rapid depolarization of serotonergic neurons, resulting in the release of a variety of neurotransmitters associated with nausea and vomiting, including dopamine, CCK, 5‐HT, etc.^12^ Surprisingly, we discovered that the 5‐HT 3A receptor decreased rather than increased at the RNA level. Recent evidence also suggested that activation of the 5‐HT 2C receptor, rather than the 5‐HT3 receptor, was involved in nausea regulation, even though the latter was of major importance for vomiting.^41^ Additionally, the activation of the 5‐HT 1A receptor was effective in inhibiting anticipatory nausea in rats by suppressing the release of 5‐HT.^43^, ^44^ These findings were consistent with our results regarding changes in 5‐HT 2C and 5‐HT 1A receptors. Notably, these differences seemed to be unique to the conditioning mechanism and were not affected by LiCl itself, implying that 5‐HT systems may play important roles in conditioned nausea.

When the nocebo occurs, significant changes in brain function are observed; however, no definitive conclusion has been reached. The available evidence is primarily based on human neuroimaging studies of nocebo hyperalgesia, and some brain regions have been suggested to be clearly activated, such as the thalamus, insula, hippocampus, ACC, PAG, amygdala, etc.^45^, ^46^, ^47^ We noted that few studies had examined changes in brain activation in nocebo nausea. Being a transcription factor encoded by the immediate early gene family, c‐Fos has been widely used as a marker of neuronal activity.^48^, ^49^ Here, we further investigated the brain maps of positive c‐Fos neurons to uncover the underlying neural mechanisms of nocebo nausea. Our findings revealed similar activated brain regions in nocebo nausea, particularly those primarily responsible for integrating cognition, emotion, and nausea messages, such as the ACC, insula, BLA, PVN, PVT, PAG, DRD, and NTS. This was in line with a recent study that detected c‐Fos expression in different brain regions after inducing conditioned nausea in male rats, with more activated neurons in the frontal cortex, insula, and PVN.^50^ The ACC and insula, the key brain regions identified in our study, could integrate emotional and cognitive stimuli to directly trigger nausea.^12^, ^51^ Strong evidence also suggested that they were associated with the production of aversion emotions.^52^, ^53^ The amygdala played an important role in the execution of emotion and motivation functions, as well as in the pairing of a conditioning stimulus with aversive or rewarding outcomes.^54^ Recent studies also reported that PVT activation pushed the establishment of the association between novel stimuli and expected punitive cues, suggesting it was important in aversive association learning.^55^ These brain activations partially explained the sustained aversion to saccharin solution in nocebo rats.

Furthermore, we confirmed that PVN had a unique effect on nausea caused by conditioning rather than observational learning. PVN is known to regulate autonomic and neuroendocrine functions, which establishes a wide connection to the limbic system, NTS, and DMV.^56^, ^57^ Oxytocin (OT) released by OT neurons in PVN, in particular, regulates a variety of neural functions, including recognition, learning, emotion, reward, and memory.^58^ Previous research found that it was critical not only in the mediation of conditioned taste avoidance but also in the expression of socially mediated conditioned nausea in rats.^59^, ^60^ More importantly, we also observed the unique changes of CCK neurons in PVN at the same time, which were not related to LiCl administrations. Additional studies are needed to investigate in more detail how PVN is involved in nocebo nausea and what role CCK plays.

As noted previously, the importance of 5‐HT systems in nausea has been shown, and we also observed that brain regions that predominantly contained serotonergic neurons, including PAG and DRD, were strongly activated. Asides from directly eliciting nausea responses, 5‐HT has recently been indicated to participate in the associative learning of conditioned and unconditioned stimulus by regulating synaptic plasticity.^61^ Somewhat unexpectedly, CCK was obviously reduced in these brain regions. It has been unclear how CCK is associated with 5‐HT systems yet, and this is a significant target for future studies. Our findings also demonstrated that nocebo nausea was accompanied by the activation of the vomiting center – NTS.^62^ Importantly, such neurobiological alterations could still be evoked after 12 days of initial nocebo nausea responses.

Some limitations should be pointed out. First, sex hormones have been suggested to broadly regulate the functions of the neural systems, including mood and cognition.^63^ Only male rats were used in our study to avoid the fluctuation of hormone levels in female rats. Although no definite conclusions could be drawn, there was some evidence that females were more susceptible to nocebo effects.^4^, ^64^ Future studies should, therefore, further assess the role of sex differences in nocebo behaviors. Second, the current rat model did not fully represent the condition of nocebo nausea seen in humans. Large differences existed between animals and humans, whether at structural or physiological levels. Humans produce the expectation of nocebo based on prior experience as well as complicated cognitive and psychological processes, while the difficulty of acquiring identical expectations has been shown in animals.^2^ Thus, the discovery revealed in the animal model needed to be further allied with human research. Third, our preliminary exploration identified the potential of CCK and related brain regions in the regulation of nocebo nausea. However, the present study cannot completely exclude the impact of the pharmacological actions of LiCl on CCK release. There was a clear need to further investigate whether and how CCK played a detailed role in developing nocebo.

## 5 CONCLUSION

In summary, the current study provides a novel experimental animal model of nocebo nausea generated by combining two distinct mechanisms of observational learning and conditioning. When rats were re‐exposed to the same environment, the associated nausea behaviors and the expectation of nausea could be evoked within 12 days. CCK was identified as the key regulator mediating nocebo nausea incidence, both peripherally and centrally. Our findings also showed the 5‐HT system and related brain areas, such as the ACC, insula, BLA, PVT, PVN, NTS, PAG, and DRD, were activated in nocebo nausea. The current animal model may aid in the development of potential nocebo molecular pathways and therapeutic strategies.

## AUTHOR CONTRIBUTIONS

Yu Zhang and Zheman Xiao participated in the study design together. Yu Zhang performed the experiments, analyzed the data, and drafted the manuscript. Wanbin Huang, Zhengming Shan, Liu Yang, and Yue Wang took responsibility for parts of the experiments. Tao Qiu and Luyu Hu extracted and recorded data on behavioral results. Yanjie Zhou took part in the statistical analysis of the data. The manuscript was modified by Yanjie Zhou, Zhengming Shan, and Zheman Xiao. All authors read and approved the final manuscript.

## FUNDING INFORMATION

This work was supported by grants from the National Natural and Science Foundation of China (81971055, 81471133, 82101292), the Natural Science Foundation of Hubei Province (No. 2020CFB226), and the Buchang Zhiyuan Research Fund (HIGHER2022094).

## CONFLICT OF INTEREST STATEMENT

The authors declared no competing interests.

## Supporting information


Data S1

## Data Availability

The data that support the findings of this study are available from the corresponding author upon reasonable request.
