# Supplementary material for: A new experimental rat model of nocebo‐related nausea involving double mechanisms of observational learning and conditioning
Source: CNS Neurosci Ther. 2023 Aug 7;30(2):e14389. doi: 10.1111/cns.14389 (PMC10848046; doi:10.1111/cns.14389)
Supplement: Supplementary file 1 — Data S1 [file CNS-30-e14389-s001.pdf]

Table S1 The sequence anf name of primers used in qPCR.

**Gene**	**Forward 5′→3′ primer**	**Reverse 5′→3′ primer**
mβ-actin	AGGCCCCTCTGAACCCTAAG	CCAGAGGCATACAGGGACAAC
CCK	CCAGGACTGCCATCACCAC	ACAGCAGCCGTTGGAAACC
CCKar	GCCTACGGGTTGATCTCTC	CTCATATCGGGTGCTGCT
CCKbr	GCCTAAGAACGGTCACCAACG	GACTGTGCCGAAGATGAATGTG
Cnr1	GGACATGGAGTGCTTTATGATTC	GAGGGACAGTACAGCGATGG
Cnr2	TGGACCTTGTGACCTTCTG	GGCTGGACTGGGATAGTG
Drd2	TGGCTGTATCCCGAGAGAA	GTAGACAACCCACGGCATT
Drd3	AGACGTGTGGCACTCATGATC	CTTTGCCTCAGGACTATGTAGA
Htr1a	GACCACGGCTACACCATCTAC	CTGTCCGTTCAGGCTCTTCTT
Htr3a	CGGGTGGGATATGTGCT	TTAGGGGACAAGGGGACT
Htr2c	CAGCCGAGTCCGTTTCT	AAGGTGTTCGTTGGCCTAT

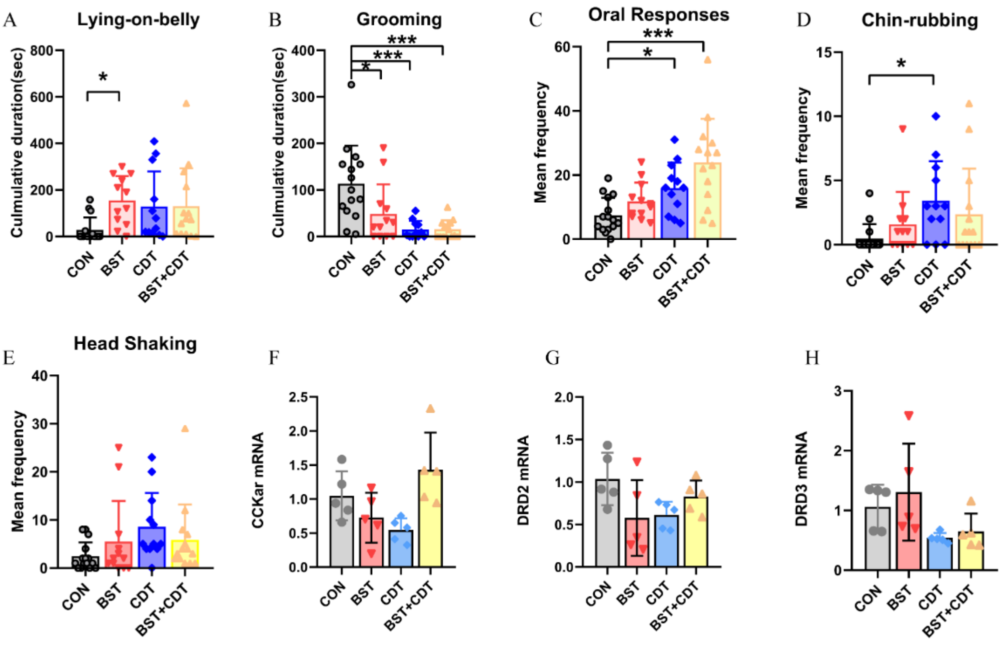

Fig.S1 Double training paradigms potentiated part of behavioral effects of nocebo nausea.

A. Comparison of lying-on belly duration under different training mechanisms; bars indicate medians with IQR, Kruskal-Wallis H test, H (3) =13.772, *P*=0.003.

B. Comparison of grooming duration under different training mechanisms; bars indicate medians with IQR, Kruskal-Wallis H test, H (3) =19.977, *P*<0.001; Generalized estimating equations showing significant main effects both of observational learning (*P*=0.019) and conditioning (*P*<0.001), and a significant interaction between them (*P*=0.021).

C. Comparison of mean frequency of oral responses under different training mechanisms; bars indicate mean with SEM, one-way ANOVA, F (3,49) =8.835, *P*<0.001.

D. Comparison of mean frequency of chin-rubbing under different training mechanisms; bars indicate medians with IQR, Kruskal-Wallis H test, H (3) =10.572, *P*=0.014.

E. Comparison of mean frequency of head shaking under different training mechanisms; bars indicate medians with IQR, Kruskal-Wallis H test, H (3) =10.095, *P*=0.018.

F-H. The RNA levels of CCKar, DRD2, and DRD3 in different experimental groups in the medulla. n=5/group; **P*<0.05, ****P*<0.001 vs. the CON group. Abbreviations: ANOVA, analysis of variance; BST, bystander; CCKar, Cholecystokinin A Receptor; CDT, conditioning; cnr, cannabinoid receptor; CON, control; DRD2, dopamine receptor D2; DRD3, dopamine receptor D3.

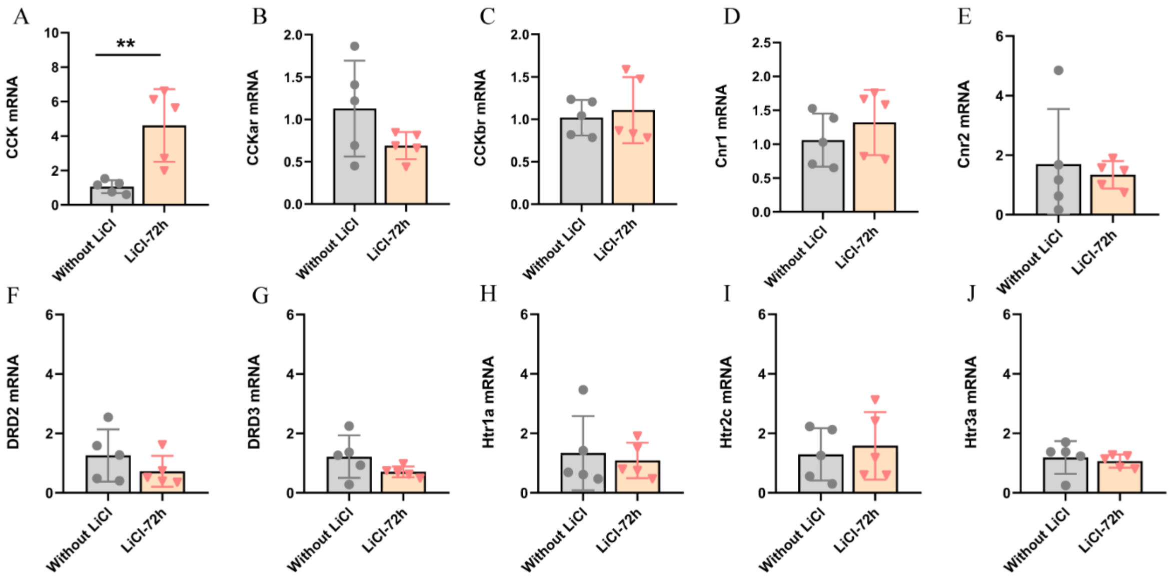

Fig.S2 LiCl alone generated an increase in the CCK mRNA level but no change in other molecules 72 h after injection.

A-J. The RNA expressions of key molecules involved in the nausea in the medulla between groups without LiCl and LiCl-72h. n=5/group; **((A)** unpaired t test, t=3.707, df=8, *P*=0.006).

Data are presented as the mean ± SEM. **P*<0.05, ***P*<0.01, ****P*<0.001; Abbreviations: ANOVA, analysis of variance; CCK, cholecystokinin; CCKar, Cholecystokinin A Receptor; CCKbr, Cholecystokinin B Receptor; 5-HT, 5-hydroxytryptamine; htr, 5-hydroxytryptamine receptor; cnr, cannabinoid receptor; DRD2, dopamine receptor D2; DRD3, dopamine receptor D3.

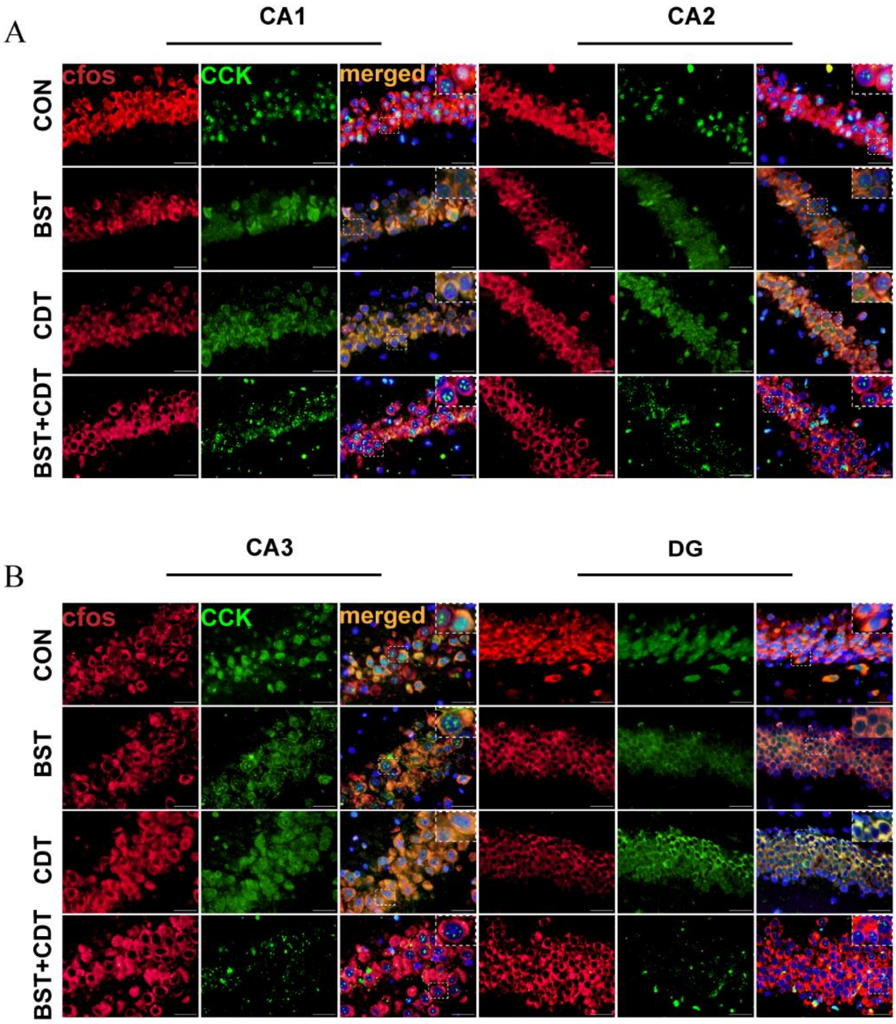

Fig.S3 The maps of c-Fos and CCK expressions in different regions of hippocampus in nocebo nausea rats.

A-B. Representative immunofluorescence staining showing the colocalization of c-Fos (red) and CCK (green) represented by orange or yellow colors. The nuclear translocation of CCK in the BST+CDT rat increased in areas of CA1, CA2 (**A**), CA3, and DG (**B**). Scale bar=20 μm. The white box was an enlarged region. Abbreviations: BST, bystander; CCK, cholecystokinin; CDT, conditioning; CON, control.

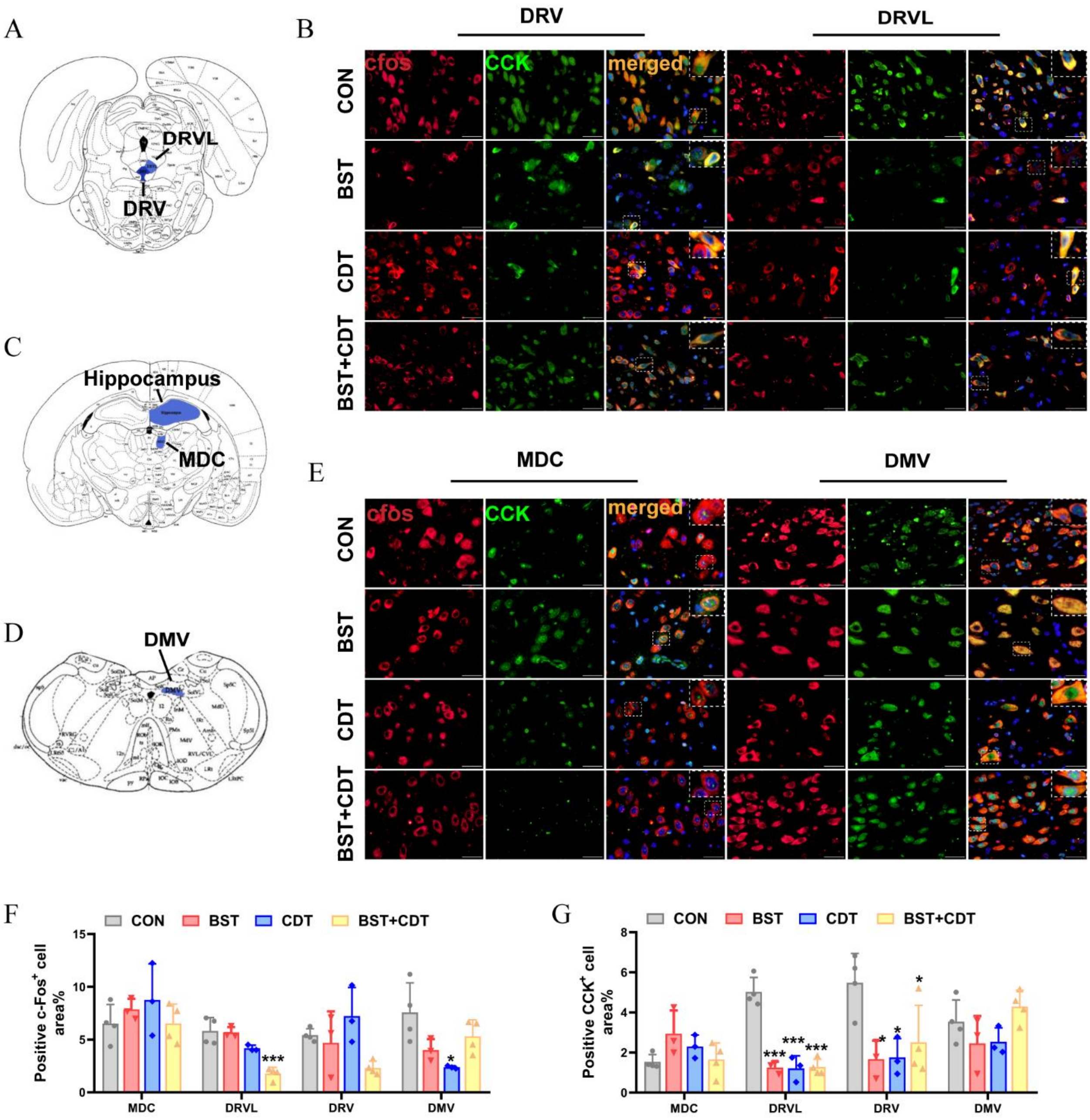

Fig.S4 Nocebo nausea did not involve in brain activations including MDC and DMV, even inhibited c-Fos and CCK expressions in DRVL and DRV.

A. Illustration of the brain atlas regarding DRV and DRVL.

B. Representative immunofluorescence staining showing decreased positive c-Fos (red) and CCK (green) neurons in DRV and DRVL. Yellow or orange colors represented the colocalization of c-Fos and CCK. Scale bar=20 μm. The white box was an enlarged region.

C-D. Illustration of the brain atlas regarding hippocampus, MDC (**C**) and DMV (**D**).

E. Representative immunofluorescence staining showing no change of c-Fos (red) and CCK (green) expressions in MDC and DMV groups. Scale bar=20 μm.

F. Quantification analysis of the area of positive c-Fos neurons in MDC, DRV, DRVL and DMV; n=3-4/group; one-way ANOVA, $F_{DRVL}(3,10) = 20.66$, $P<0.001$, $F_{DMV}(3,10) = 5.053$, $P=0.021$.

F. Quantification analysis of the area of positive CCK neurons in MDC, DRV, DRVL and DMV; n=3-4/group; one-way ANOVA, F_{DRVL} (3,10) =45.07, $P<0.001$, F_{DRV} (3,10) =5.992, $P=0.014$.

Data are presented as the mean ± SEM; *$P<0.05$, **$P<0.01$, **$P<0.001$ vs. the CON group. Abbreviations: ANOVA, analysis of variance; BST, bystander; CCK, cholecystokinin; CDT, conditioning; DMV, dorsal motor nucleus of the vagus; CON, control; DRV, dorsal raphe nucleus-ventral part; DRVL, dorsal raphe nucleus-ventrolateral part; MDC, mediodorsal thalamus-central subnucleus.

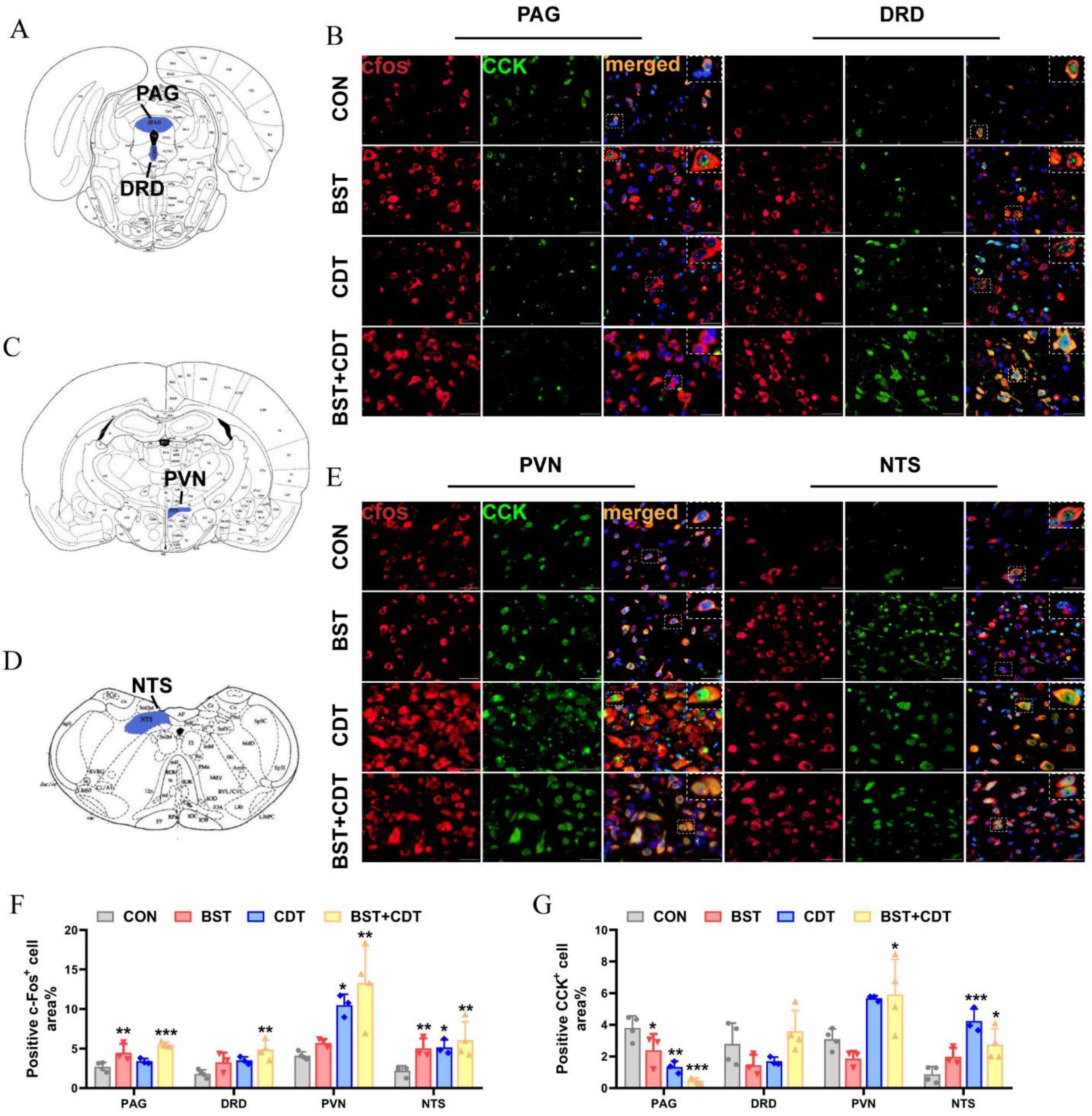

Fig.S5 Nocebo nausea activated c-Fos neurons in brain regions related to nausea afferent signals, which were accompanied by changes in CCK expression.

A. Illustration of the brain atlas regarding PAG and DRD.

B. Representative immunofluorescence staining showing increased positive c-Fos (red) neurons in PAG and DRD,

with CCK (green) expression inhibited in PAG. Yellow or orange colors represent the colocalization of c-Fos and CCK. Scale bar=20 μm. The white box was an enlarged region.

C-**D**. Illustration of the brain atlas regarding PVN (**C**) and NTS (**D**).

E. Representative immunofluorescence staining showing increased positive c-Fos (red) and CCK (green) neurons in PVN and NTS. Scale bar=20 μm.

F. Quantification analysis of the area of positive c-Fos neurons in PAG, DRD, PVN and NTS; n=3-4 /group; one-way ANOVA, $F_{PAG}(3,10)=14.02$, $P<0.001$, $F_{DRD}(3,10)=7.702$, $P=0.006$, $F_{PVN}(3,10)=9.438$, $P=0.003$, $F_{NTS}(3,10)=5.025$, $P=0.022$.

G. Quantification analysis of the area of positive CCK neurons in PAG, DRD, PVN and NTS; n=3-4 /group; one-way ANOVA, $F_{PAG}(3,10)=19.05$, $P<0.001$, $F_{PVN}(3,10)=8.086$, $P=0.005$, $F_{NTS}(3,10)=12.39$, $P=0.001$.

Data are presented as the mean ± SEM; *$P<0.05$, **$P<0.01$, ***$P<0.001$ vs. the CON group. Abbreviations: ANOVA, analysis of variance; BST, bystander; CCK, cholecystokinin; CDT, conditioning; CON, control; DRD, dorsal raphe nucleus-dorsal part; NTS, nucleus tractus solitarius; PAG, periaqueductal gray; PVN, hypothalamic paraventricular nucleus.

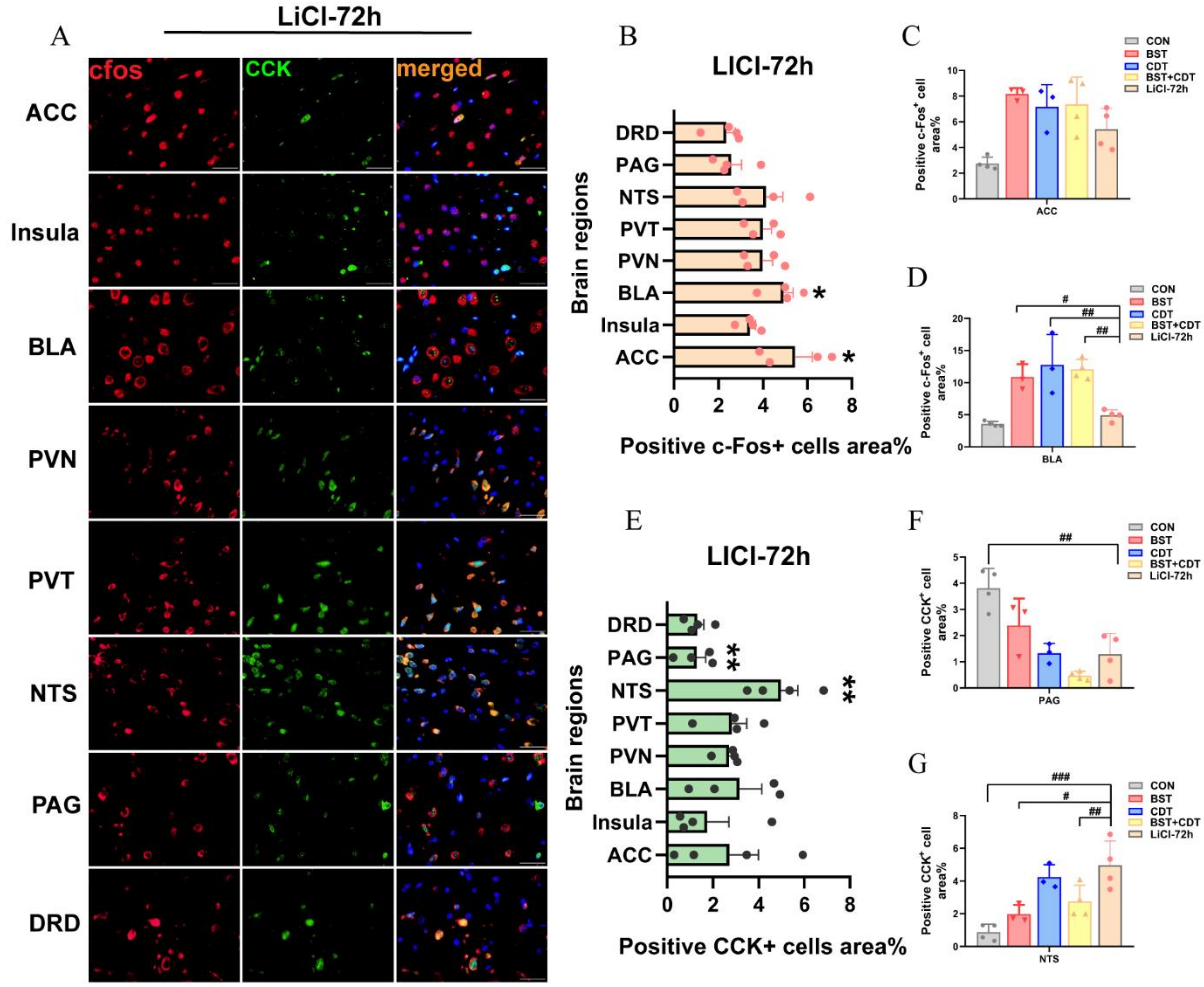

Fig.S6 ACC and BLA were activated after 72 hours of LiCl administration, with CCK expressions changing in PAG and NTS.

A. Representative immunofluorescence staining in nocebo-nausea related brain regions after 72 hours of LiCl administration. c-Fos (red), CCK (green), and the colocalization of c-Fos and CCK (yellow or orange). Scale bar=20 μm.

B. Quantification analysis of the area of positive c-Fos neurons in different brain regions; n=4/group, compared to the CON group, unpaired t test, t_{ACC} =4.265, df=6, *P*=0.005, and t_{BLA} =2.783, df=6, *P*=0.032.

C. Comparison of the area of positive c-Fos neurons in the ACC among LiCl-72h and other experimental groups.

D. Comparison of the area of positive c-Fos neurons in the BLA among LiCl-72h and other experimental groups; one-way ANOVA, F (4,13) =14.03, *P*<0.001.

E. Quantification analysis of the area of positive CCK neurons in different brain regions; n=4/group, compared to the CON group, unpaired t test, t_{PAG}=,4.586 df=6, *P*=0.004, and t_{NTS} =5.270, df=6, *P*=0.002.

F. Comparison of the area of positive CCK neurons in the PAG among LiCl-72h and other experimental groups; one-way ANOVA, F (4,13) =13.71, *P*<0.001.

G. Comparison of the area of positive CCK neurons in the NTS among LiCl-72h and other experimental groups; one-way ANOVA, F (4,13) =11.22, *P*<0.001.

Data are presented as mean ± SEM; **P*<0.05, ***P*<0.01, ****P*<0.001, #*P*<0.05, ##*P*<0.01, ###*P*<0.001; Abbreviations: ANOVA, analysis of variance; ACC, anterior cingulate; BLA, basalateral amygdala; BST, bystander;

CCK, cholecystokinin; CDT, conditioning; CON, control; DRD, dorsal raphe nucleus-dorsal part; NTS, nucleus tractus solitarius; PAG, periaqueductal gray; PVN, hypothalamic paraventricular nucleus; PVT, thalamic paraventricular nucleus.
